# Pancreatic schwannoma- CT and MRI findings: A rare case report and review of literature

**DOI:** 10.1016/j.amsu.2021.102664

**Published:** 2021-08-05

**Authors:** Narjisse Aichouni, Widad Abbou, Siham Nasri, Wafaa Khannoussi, My Zahi Ismaili, Tijani El Harroudi, Amal Bennani, Imane kamaoui, Imane Skiker

**Affiliations:** a Radiology Department, Mohammed VI University Hospital, Faculty of Medicine and Pharmacy, University Mohammed I, Oujda, Morocco; b Gastroenterology Department, Mohammed VI University Hospital, Faculty of Medicine and Pharmacy, University Mohammed I, Oujda, Morocco; c Surgery Oncology, Mohammed VI University Hospital, Regional Oncology Center, Faculty of Medicine and Pharmacy, University Mohammed I, Oujda, Morocco; d Pathology Department, Mohammed VI University Hospital, Faculty of Medicine and Pharmacy, University Mohammed I, Oujda, Morocco

**Keywords:** Schwannoma, Tumor, Pancreatic mass, Computed tomography, Case report

## Abstract

**Introduction:**

Pancreatic schwannoma (PS) is an extremely rare benign tumor. Here we describe the Computed Tomography (CT) and Magnetic Resonance Imaging (MRI) results of PS in a 59 years old woman, as well as a review of the literature.

**Case presentation:**

A 59-year-old woman consulted for atypical epigastralgia without fatigue, weight loss or fever. CT scan and MRI showed a 35 mm inhomogeneous lesion with well-defined margins located in the pancreas head. The diagnosis of pancreatic tumor was made. The pathologic examination of the biopsied mass yielded a diagnosis of pancreatic schwannoma.

**Clinical discussion:**

On CT scans, almost all benign PS are well-defined cystic or low-density masses. MRI is helpful in characterizing their typical encapsulation.

**Conclusion:**

The detection of pancreatic schwannoma is extremely rare. Although multiple imaging modalities are currently available, it is challenging to make an accurate diagnosis before operation.

## Highlights

• Although multiple imaging modalities are currently available, it is challenging to make an accurate diagnosis before operation.
• Pancreatic schwannomas of Antoni type A show solid masses with inhomogeneous enhancement. Those of Antoni type B show homogeneous cystic or multiseptated mass.
• Schwannoma must be discussed as one possibility in the list of differential of pancreatic neoplasms.

## 1 Introduction

Pancreatic schwannomas (PS) are uncommon mesenchymal tumors that develop from Schwann cells in the sheaths of peripheral nerves [1]. Although tumors can occur in any part of the body, the most common parts are the extremities, trunk, head and neck, in which histopathology is the main diagnostic tool for tumors in unusual sites [2,3]. Despite the use of various imaging modalities, pancreatic schwannomas preoperative diagnosis is extremely challenging. To the best of our knowledge, in the English literature less than 70 PS cases have been documented in the last 30 years. We describe a pancreatic schwannoma case and conduct a literature review with an emphasis on CT and MRI findings in this report. This case report has been reported in line with the SCARE Criteria [4].

## 2 Presentation of case

**Fig. 1:**
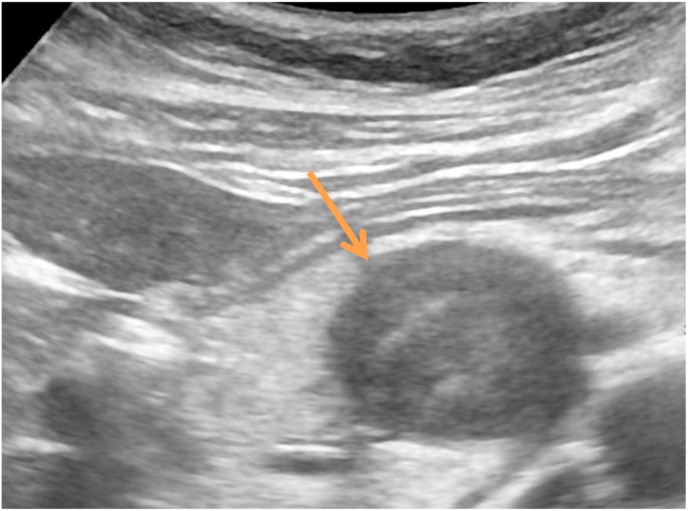
Abdominal ultrasound revealing a round lesion with a uniform low echo at the pancreas's head.

**Fig. 2:**
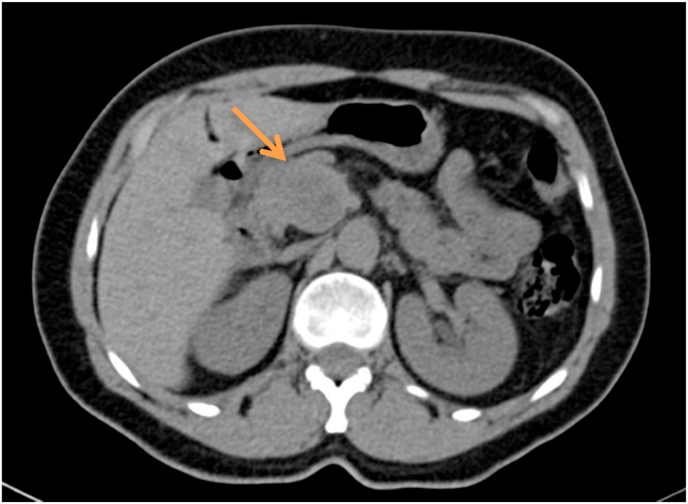
Spiral CT of the abdomen before contrast administration reveals a 35mm hypodense lesion with well-defined borders in the pancreas's head (arrow).

**Fig. 3:**
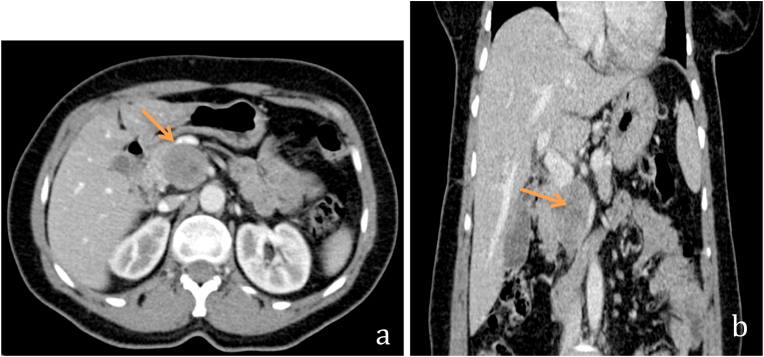
Axial (a) and coronal (b) sections of contrast-enhanced CT scan of the abdomen showing a focal mass with inhomogeneous enhancement in the pancreatic head (arrow).

**Fig. 4:**
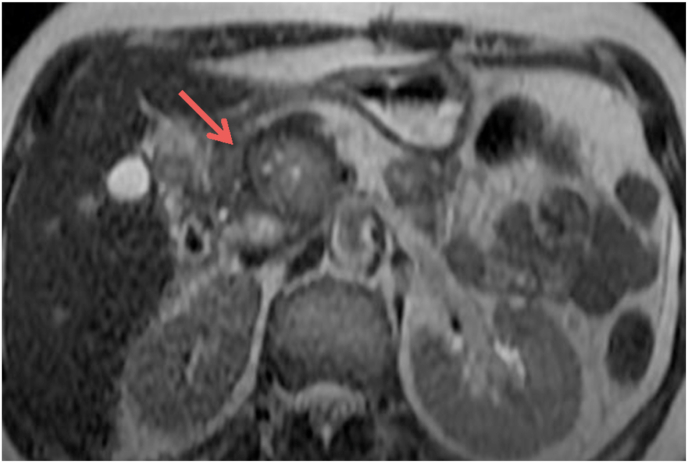
MRI a T2-weighted axial sequence showing an encapsulated mass with well-defined borders located in the pancreas's head (arrow).

A 59 years old woman, married with no previous pathological history and without surgical history or toxic habits, consults for atypical epigastralgia that is not accompanied by fever, fatigue or weight loss. She denied having ever had pancreatitis. The physical examination was completely normal. CA19-9 tumor serum markers and carcinoembryonic antigen were both negative. An abdominal ultrasound revealed a tiny rounded lesion with low uniform echo at the pancreas's head (Fig. 1). The abdomen Spiral CT prior to contrast medium administration revealed in the pancreas's head a 35mm hypodense lesion with well-defined borders (Fig. 2). The lesion showed modest inhomogeneous enhancement on multiple-phase enhanced scans after a bolus injection of intravenous contrast medium (Fig. 3). The lesion had an intimate contact with the superior mesenteric artery without peripheral lymphadenopathy or distant secondary lesions. The diagnosis of pancreatic tumor was made. The mass was preliminarily considered as a solid pseudopapillary tumor of the pancreas. We also discussed several types of tumor including a serous cystadenoma, a neuroendocrine tumor and a pancreatic schwanoma. MRI was then performed to better analyze the tumor, revealed an encapsulated mass with well-defined margins (Fig. 4). Moreover, there were no dilatation and calcification of the duct of the pancreas.

After an echoendoscopy biopsy yielded inconclusive results, a surgical open biopsy under general anesthesia was performed. The pancreatic mass's histopathology revealed spindle cells organized in a fascicular manner. The cells have elongated nuclei sometimes with an angular aspect. A pathologic testing showed that the patient had pancreatic schwannoma. In addition, immunohistochemical staining showed that for S-100, tumor cells were positive. Given the surgical risk (intimate contact with the superior mesenteric artery) and the patient's non-consent for the surgery, a decision of close monitoring was decided. At the 4-month follow-up visit, the patient was doing well.

## 3 Discussion

Schwannoma, also known as neurilemmoma, is a benign tumor that develops from schwann cells found in the sheaths of cranial, spinal nerve roots, and peripheral nerves. Pancreatic schwannomas are rare neoplasms that arise from the vagus nerve branches that run through the pancreas [5]. PS is most commonly seen in people at varying ages with no gender preponderance. Patients with PS typically appear with non specific abdominal pains [6]. Benign neurogenic tumors account for 65% of all neurogenic tumors, although 10–15% are malignant [7]. The bulk of these tumors were observed in the pancreas's head (42.3%) and the pancreas's body (23%) [8].

Classical PS tend to be divided into two distinct areas under the microscope: Antoni A and Antoni B. A hypercellular zone of closely packed, long bipolar cells (spindle cells) organized in interlacing and palisading styles characterizes the AntoniA area. There are also verocaybodies lacking mitotic figures. On the other hand, loose hypocellular areas with degenerative alterations such as hemorrhage, calcification, hyalinization, xanthoma infiltration, and cyst development characterize the Antoni B area [9].

Histopathological examination is mostly used to determine the type of tumor. The presence of positive proteins S100, Vimentin, and CD56 in the immunohistochemistry study confirms the presence of Schwannoma. CD57 and GFAP are occasionally expressed in schwannomas, whereas cytokeratin AE1/3, CD34, c-kit, desmin, and myosin are not [10].

The preoperative diagnosis of PS is difficult due to its rarity and non specific imaging appearance. Appearance of solid and cystic areas in these tumors depends on the proportion of Antoni A and B areas [7].

Diagnosis of asymptomatic PS patients is difficult and Computed tomography scan is helpful to deliniate the anatomic relationship between the tumor and surrounding tissues. Well-defined round masses with numerous, low-attenuation cystic necrotic areas are the most common CT appearance of benign PS. Antoni type A patients have hypodense, solid masses with inhomogeneous or rarely multiseptated enhancement. Antoni type B are homogeneous cystic or multiseptated masses. Moreover, a previous research discovered that intravascular thrombosis occurs in Antoni B areas, causing necrosis and the development of cysts [11]. Previous findings indicated that the size of pancreatic schwannomas is (1.5–20 cm), cystic tumors (3.0–20 cm) and solid tumors (1.5–3.5 cm) [12]. After the administration of a contrast agent, Antoni A and Antoni B components of the tumor can be well distinguished from each other on the CT scan. Antoni A areas are usually enhancing lesions where as Antoni B areas are frequently non enhancing lesions. Therefore, Antoni A areas are often more vascular than Antoni B areas [13].

Well-defined round masses with numerous, low-attenuation cystic necrotic areas are the most common CT findings of benign pancreatic schwannomas. Malignancy is indicated by rapid development, invasion of surrounding tissues, strong contrast enhancement of a solid inhomogeneous mass with irregular contours, and vascular thrombosis [12].

Our patient's CT scan revealed a discretely and inhomogeneously increased mass that corresponded exactly to the scannographic appearance of PS Antoni type A reported in the literature.

On MRI, a typical schwannoma appears encapsulated, hypointense in T1-weighted images and appears inhomogeneously hyperintense in T2-weighted images [14]. Magnetic resonance imaging (MRI) can usually outline the degree of vascular involvement of the tumor, which may be greatly helpful in differentiating the potential biological behavior of the lesion in terms of being benign or malignant [15].

In our case, MRI was performed to better analyze the pancreatic lesion and the pancreatic ducts. It revealed an encapsuled mass, hypointense in T1 and hyperintene heterogeneous in T2 with no calcification or dilation of the ducts of the pancreas.

In the cystic neoplasms or pseudocysts differential diagnosis, cystic pancreatic schwannomas must be considered, but islet cell tumors, carcinoma, or other rare benign tumors like papillary and solid neoplasms are considered for differentiation from solid PS. Histopathologic examination is used to get the exact and final diagnosis.

Location and locoregional involvement guide PS management. For benign schwannomas, simple enucleation is the best treatment option. To accomplish R0 resection in tumors with malignant characteristics, pancreatoduodenectomy or distal pancreatectomy with/without splenectomy may be required. Intraoperative frozen sections may be useful in determining whether a schwannoma is benign or malignant, hence avoiding extensive surgical resection and accompanying morbidity [4].

## 4 Conclusion

Dispete its rarity, schwannoma must be discussed as one possibility in the list of differential of pancreatic neoplasms. The imaging finding plays a critical role in the accurate diagnosis of this diseases. The rapid evolution, vascular encasement, or visceral invasion should elicit suspicion of malignant transformation.

## Ethical approval

The ethical committee approval was not required given the article type (case report).

## Sources of funding

None.

## Author contribution

Aichouni Narjisse: study concept, data analysis; writing review & editing. Widad Abbou: Study conception, data analysis. Nasri Siham: contributor. Wafaa Khannoussi: contributor. My Zahi Ismaili: contributor. Tijjani El Harroudi:. Contributor. Amal Bennani: contributor. Imane Kamaoui: contributor. Imane Skiker: Supervision and data validation.

## Registration of research studies

As this manuscript was a case report with no new medical device nor surgical techniques, not prior registration is required.

## Guarantor

Narjisse Aichouni.

## Provenance and peer review

Not commissioned, externally peer-reviewed.

## Consent

Written informed consent was obtained from the patient for publication of this case report and accompanying images. A copy of the written consent is available for review by the Editor-in-Chief of this journal on request.

## Declaration of competing interest

The authors state that they have no conflicts of interest for this report.

## References

[1] Xu Shao-Yan, Sun Ke, Owusu-Ansah KwabenaGyabaah, Xie Hai-Yang, Zhou Lin, Zheng Shu-Sen, Wang Wei-Lin (2016). Central pancreatectomy for pancreatic schwannoma: a case report and literature review. World J. Gastroenterol..

[2] Wang S, et al. Pancreatic schwannoma mimicking pancreatic cystadenoma: a case report and literature review of the imaging features. Medicine (Baltim.). 2019 Jun;98(24):e16095. doi: 10.1097/MD.0000000000016095. PMID: 31192973; PMCID: PMC6587594.). 10.1097/MD.0000000000016095 PMC6587594 31192973

[3] Eldsoky I, et al. The predictive value of nasolacrimal sac biopsy in endoscopic dacryocystorhinostomy. Ann Med Surg (Lond). 2021 Apr 16;vol. 65:102317. doi: 10.1016/j.amsu.2021.102317. PMID: 33981427; PMCID: PMC8085898. 10.1016/j.amsu.2021.102317 PMC8085898 33981427

[4] Agha R.A., Franchi T., Sohrabi C., Mathew G., for the SCARE Group (2020). The SCARE 2020 guideline: updating consensus surgical CAseREport (SCARE) guidelines. Int. J. Surg..

[5] Devi J., SathyalaKShmi R., ChanDRamouleeSwaRi K., SumitRa Devi nalli R. (2014). Pancreatic schwannoma - a rare case report. J. Clin. Diagn. Res..

[6] Kumar Varshney Vaibhav, Elhence Poonam, Sureka Binit, Taruna Yadav (2020). Preoperative diagnosis of pancreatic schwannoma – myth or reality. J. Canc. Res. Therapeut..

[7] Tofigh ArashMohammadi, Hashemi Mohammad, NematiHonar Behzad, Solhjoo Fereidoon (2008). Rare presentation of pancreatic schwannoma: a case report. J. Med. Cases.

[8] Kinhal Vidyadhar A., Ravishankar T.H.S., Melapure Ashok I., Jayaprakasha G., Manjunath Range Gowda B.C. (2010). Pancreatic schwannoma: report of a case and review of literature. Indian J. Surg..

[9] Han Wanga, Zhangb Bing-Bing, Wangc Shen-Fan, Zhongd Jing-Jiao, Zhengb Jian-Ming (2020). Huan Han. Pancreatic schwannoma: imaging features and pathological findings. Hepatobiliary Pancreat. Dis. Int..

[10] Witkowski Grzegorz, Kołos Małgorzata, Nasierowska-Guttmejer Anna, Durlik Marek (2020). Neuroma (schwannoma).A rare pancreatic tumor. Pol. Przegl. Chir..

[11] Hanaoka Taro, Okuwaki Kosuke, Imaizumi Hiroshi, Imawari Yusuke (2021). Pancreatic schwannoma diagnosed by endoscopic ultrasound-guided fine-needle aspiration. Intern. Med..

[12] Yu R.S., Sun J.Z. (2016). Pancreatic schwannoma: CT findings. Abdom. Imag..

[13] Abu-Zaid A., Azzam A., Abou Al-Shaar H., Alshammari A.M., Amin T., Mohammed S. (2013). Pancreatic tail schwannoma in a 44-year-old male: a case report and literature review. Case Reports in Oncological Medicine.

[14] Ma Y., Shen B., Jia Y., Luo Y., Tian Y., Dong Z. (2017). Pancreatic schwannoma: a case report and an updated 40-year review of the literature yielding 68 cases. BMC Cancer. 14 déc.

[15] Shi Zenshan (2021). MR imaging features of pancreatic schwannoma. A Chinese case series and a systematic review of 25 cases. Cances imaging.

